# Use of Remote Consultations by Chiropractors in the United Kingdom During the COVID-19 Pandemic: A Cross-Sectional Survey

**DOI:** 10.1016/j.jcm.2025.07.003

**Published:** 2025-10-01

**Authors:** Marc W. Sanders, Jonathan Field, Dave Newell, Neil Osborne

**Affiliations:** a AECC School of Chiropractic, Health Sciences University, Bournemouth, United Kingdom; b Centre for Primary Care and Population Studies, University of Southampton, Southampton, United Kingdom

**Keywords:** Chiropractic, Surveys and questionnaires, COVID-19, Coronavirus, Telemedicine, Musculoskeletal pain

## Abstract

**Objectives:**

The purpose of this study was to explore the use of remote consultations (RCs) by chiropractors in the United Kingdom.

**Methods:**

All registered practicing UK chiropractors were invited to participate in an online survey during part of the first UK national lockdown period of the COVID-19 pandemic (May 2020 to June 2020). This survey collected information on 1) chiropractor demographics, 2) use of RCs by chiropractors, and 3) chiropractors’ views of RCs. Descriptive and inferential statistics (chi-squared and Spearman’s Rho) were used to analyze the data.

**Results:**

The response rate was 17.1% (534/3131). A third of respondents (32.5%) had been using RCs (telephone) prior to the pandemic. Two-thirds (67.2%) used RCs during the first lockdown period and included uptake of video consultations (6.6%), phone consultations (30%), or a combination of video and phone (30.7%). A majority (58.6%) responded that they planned to continue RCs after practice restrictions were lifted. Under half (47.8%) gave their opinion that RCs would not be as effective as face-to-face care, a similar proportion (50.1%) stated they were engaging their patients with active care more than typical. Only around a half of the respondents (52.5%) were confident in carrying out RCs.

**Conclusions:**

This survey provides preliminary data on RCs delivered by UK chiropractors - a traditionally ‘hands-on’ profession. Both telephone and video RCs increased during the first UK national lockdown but confidence in carrying out RCs and impressions of their effectiveness was mixed.

## Introduction

Musculoskeletal (MSK) conditions are the United Kingdom’s (UK) leading cause of disability for years lived with disability (YLDs) and third leading cause for disability-adjusted life years (DALYs).^1^^,^^2^ The advent of contemporary communications technology has facilitated a variety of new approaches to healthcare. Telehealth is defined as health care provided remotely to a patient in a separate location using 2-way voice or visual communication, such as by computer or cell phone.^3^

**Fig. 1:**
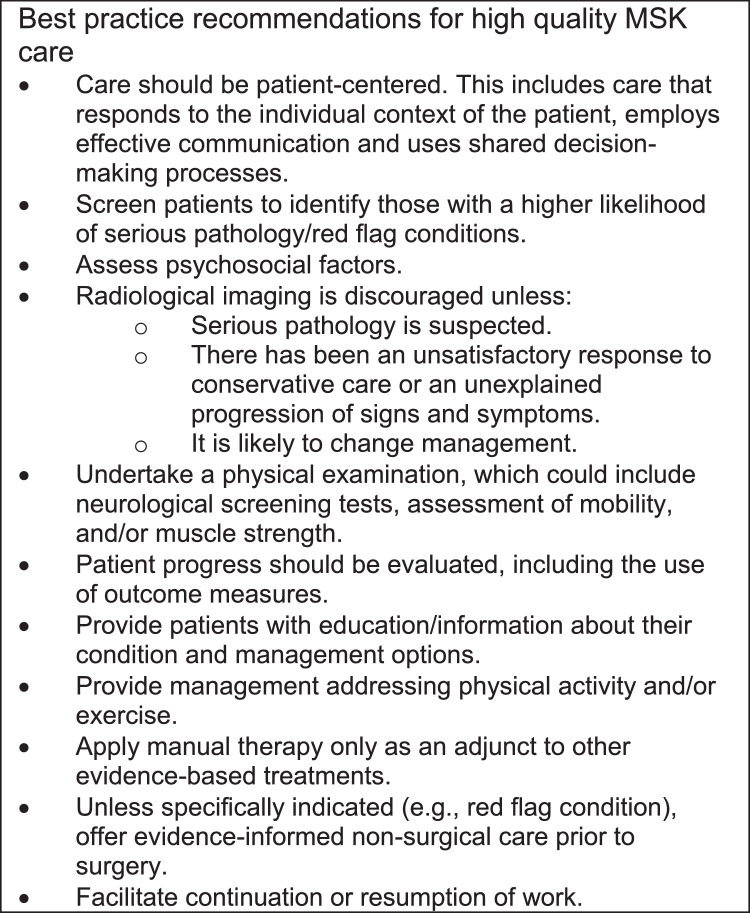
Best practice recommendations for high quality MSK care. Note: A list of 11 best practice recommendations for high quality MSK care. The above figure was adapted from Lin I, Wiles L, Waller R, et al. What does best practice care for musculoskeletal pain look like? Eleven consistent recommendations from high-quality clinical practice guidelines: systematic review. *British journal of sports medicine.* 2020;54(2):79-86.

Telehealth, includes the ability for clinicians to communicate with patients remotely via voice or video in remote or virtual consultation (RC) across a wide variety of healthcare fields including MSK healthcare that is provided by clinicians such as chiropractors, osteopaths, and physiotherapists. Whilst the literature concerning such approaches has highlighted implementation challenges, the interest, use, and subsequent research is rapidly increasing.^4^ Importantly, RCs can be aligned with best practice recommendations including “offering manual therapy only as an adjunct to other treatments” and “undertaking a physical examination” being less than amenable to implement (Fig. 1).^5^ During the COVID-19 pandemic the use of RCs brought potential benefits to preventing the virus spread, by removing the need for face-to-face contact and reducing patient travel, both key risks in viral transmission. UK chiropractic professional organizations swiftly disseminated advisory practice guidelines including the use of RCs and chiropractors were advised to only see patients face-to-face in exceptional circumstances, based on an urgent clinical need.^6^, ^7^, ^8^ This reflected the guidance for UK general medical practitioners at the time, which rapidly reconfigured protocols to minimize face-to-face appointments with patients to reduce the risk of infection.^9^

Whilst there has been a recent surge in the use of RCs in UK general practice (GP) and the physical therapies as a rapid response to adapting usual care during the COVID-19 crisis, historically the uptake for this form of healthcare delivery has been low.^10^, ^11^, ^12^, ^13^, ^14^, ^15^, ^16^ Usage level data of telephone consultations derived from routine consultation data from GP in the UK were low (18%) compared to face-to-face consultations, despite their adoption doubling from 2007 to 2014.^17^^,^^18^

Clinician attitudes towards a new health intervention is amongst potential barriers that influence their adoption and sustained use within healthcare systems.^19^, ^20^, ^21^, ^22^, ^23^, ^24^ This is exemplified by UK GPs who have explained the lack of engagement with telehealth in GP. Such reasons include, amongst other concerns, that RCs would be detrimental to clinical practice as the information they gained via such consultations could not provide a sufficient level of diagnostic accuracy, and GPs were also concerned about patient security, technical issues, and a perceived increase in workload.^15^

The experiences and perspectives of patients utilizing telehealth has been explored in the literature.^25^, ^26^, ^27^, ^28^, ^29^, ^30^, ^31^, ^32^, ^33^, ^34^ Remote consultations have been shown to be associated with high patient and staff satisfaction and similar outcomes compared to face to face consultations in GP.^4^ More specifically, remote consultations for spinal pain and other MSK complaints have been shown to reduce pain and disability and to be associated with high levels of patient satisfaction.^35^, ^36^, ^37^, ^38^

Telehealth assessment of patients with MSK complaints has been shown to be reliable and feasible compared to traditional face-to-face assessments.^38^ Several studies found that the validity and reliability of telehealth assessments of patients with MSK complaints can vary from moderate to excellent when compared to face-to-face assessments.^39^^,^^40^ However, the validity and reliability varied depends on the type of assessment; there was shown to be good validity for assessing pain, swelling, range of motion, muscle strength, balance, gait and functional assessment, but other assessments including lumbar spine posture, special orthopedic tests, neurodynamic tests and scar assessments had low to moderate validity.^38^, ^39^, ^40^ In addition, the risk of bias of current diagnostic reliability and validity studies varies from high to low when assessed by quality appraisal tools, and the certainty of evidence assessed by the GRADE approach varies from very low to high depending on the assessment type.^38^, ^39^, ^40^, ^41^

Experiences of clinicians using telehealth have been explored across a range of healthcare fields as diverse as psychotherapy, treatment of mild traumatic brain injury, and within GP.^17^^,^^24^^,^^30^^,^^42^^,^^43^ However, prior to 2020 studies of the usage of RCs within the physical therapy professions was very limited and the authors were unable to find studies involving the usage or experience of RCs by chiropractors or osteopaths in the UK at the time of the first UK national lockdown. A limited number have been published by the chiropractic profession since 2020.^44^, ^45^, ^46^, ^47^, ^48^, ^49^, ^50^, ^51^, ^52^, ^53^, ^54^, ^55^, ^56^, ^57^

The restrictions in place during the first UK national lockdown to prevent the spread of COVID-19 rapidly motivated the interest in the use of RCs amongst professions historically defined by face-to-face and/or hands on care. The use of telehealth and a "RC first" approach may become an important part of chiropractic practice and potentially all healthcare settings in the future.

Therefore, the purpose of this study was to describe the frequency and attitudes toward remote consultations to deliver patient care amongst UK based chiropractors. The aim of the survey design was to collect data to cover a broad set of constructs that are commonly reported in telehealth research whilst limiting the number of questions to minimize participant time and effort which can leverage higher response rates and thus greater representation of the profession.^58^

This report is 1 of 2 reports derived from a single cross-sectional survey,^59^ the second report addresses the qualitative aspects of the survey, specifically the responses to a free text box question, which is detailed in Supplemental file 1.

Research questions were;

• What is the frequency and pattern of past, present, and intended future use of phone, video, and combined phone and video remote consultations by chiropractors during part of the first UK national lockdown period of the COVID-19 pandemic in the UK?
• What are the views of these chiropractors concerning this approach amongst those who use and do not use remote consultations?

## Methods

### Design

The study was a national, cross-sectional survey and adhered to the recommendations of STROBE for cross-sectional survey reporting.^60^^,^^59^

### Setting

The study was conducted online and restricted to UK chiropractors from May 2020 to June 2020.

### Participants and Survey Dissemination

All UK chiropractors registered as practicing with the General Chiropractic Council (GCC) were eligible to participate in this study. UK chiropractors were recruited with the consent of their UK chiropractic professional bodies and associations (Royal College of Chiropractors (RCC), British Chiropractic Association (BCA), United Chiropractic Association (UCA), McTimoney Chiropractic Association (MCA), and Scottish Chiropractic Association (SCA)) and the UK regulatory body (GCC), who disseminated the email to all their members. Recruitment was also conducted via social media posts in private closed chiropractic social media (Facebook) groups, and by group posts and an email in a private chiropractic collaboration platform (Slack).

UK chiropractors who were registered with the GCC as non-practicing were not eligible to take part in this study. The email contained a *Participant Information Sheet* and contained details of the proposed research, confidentiality/data protection information, informed consent, author contact details and a link to the online survey. No unique identifiers were used to ensure anonymity of study participants. Participants were instructed to complete the survey only once.

### Ethics

This study received approval from the AECC University College Ethics Sub Committee on the 13/05/2020 (#E124/05/2020). Participants were informed within the survey introductory text that their consent to take part in the research study and use of their anonymized data is implied by submitting a completed survey.

### Sample Size

The target population was all GCC registered chiropractors in the UK (n = 3,355 as of 28th May 2020 (personal communication with the GCC)) as contacted by the GCC who disseminated the survey. Typical return rates for surveys in the chiropractic profession have been reported to lie between 10% and 87%.^61^ Given the reasonable assumption that all GCC registered chiropractors received an email to access the survey and assuming the lowest reported return rate of 10% (n=330) we estimated this sample size would achieve a 5% margin of error with 95% confidence.

### Survey

A questionnaire was constructed by the authors (Supplemental file 1) for the purposes of this study and was not subjected to any formal validation procedure. The questions were generated *de novo* from themes in the medical eHealth literature.^62^, ^63^, ^64^, ^65^, ^66^^,^^58^^,^^67^

The data were collected via anonymous online questionnaire using Google Forms and exported into an Excel spreadsheet. The questionnaire was comprised of 3 parts; demographic data, usage data, and chiropractic perceptions and views data. The online survey included 4 questions concerning participant demographics, 5 questions on usage of RCs, and 3 questions on views of RCs. The questions were grouped in sections by topic and where appropriate Likert scales were arranged in descending order.

### Data Analysis

Survey responses were analyzed descriptively for each question within the survey sections. Data were reported as percentages or frequencies. For scales constituting strongly agree, agree, undecided, disagree and strongly disagree we collapsed into those that agreed, were undecided, or disagreed. For inferential analysis including differences or correlations between categories, chi-squared tests for trend or Spearman’s Rho statistics were used respectively.

## Results

Overall, 534 UK registered chiropractors responded to the survey which constitutes 17.1% of registered practicing chiropractors (n = 3131). Eighteen duplicates were excluded by identifying identical free text comments. Of the 534 respondents, the majority fall between 30 and 59 years of age with the largest proportion having been in practice for 16-20 years. Just over half are female and the majority of the sample were members of the BCA (62.0%) (Table 1).

**Table 1:** Description of Respondent Characteristics

Demographic variable	n(%)
**Age**	
21-29	62 (11.6)
30-39	147 (27.5)
40-49	146 (27.3)
50-59	129 (24.2)
60-79	50 (9.4)
**Sex**	
Female	280 (52.4)
**Association**	
BCA	331 (62.0)
MCA	64 (12.0)
UCA	68 (12.7)
SCA	40 (7.5)
Other	31 (5.8)
**Years in practice**	
0-1 years	16 (3.0)
2-5 years	71 (13.3)
6-10 years	100 (18.8)
11-15 years	87 (16.3)
16-20 years	111 (20.8)
21-30 years	97 (18.2)
31-40 years	43 (8.1)
41-50 years	8 (1.5)

BCA, British Chiropractic Association; MCA, McTimoney Chiropractic Association; UCA- United Chiropractic Association; SCA, Scottish Chiropractic Association.

Nearly 70% of respondents had not used RCs before COVID-19. However, at the time of the survey this figure reversed to nearly 70% adopting or planning to adopt some form of RC use (Table 2). 41.1% indicated they were probably or definitely not planning to use RCs post COVID-19. However, a significant proportion strongly (30.9%) or moderately (27.8%) indicated they would use some form of RC after the pandemic had passed.

**Table 2:** Use of Remote Consultations

Variable	Phone	Video	Phone/Video	None	Total responded
	*n (%)*	*n (%)*	*n (%)*	*n (%)*	*n (%)*
**Pre COVID-19**	142 (27.8)	5 (1.0)	19 (3.7)	344 (67.5)	510 (95.5)
**Current**	160 (30.0)	35 (6.6)	164 (30.7)	175 (32.8)	534 (100.0)
**Planning to**	97 (19.9).	45 (9.2)	196 (40.2)	150 (30.7)	488 (91.4)

**Post COVID-19**					**Total responded**
*Definitely*	*Very probably*	*Possibly*	*Probably not*	*Definitely not*	*n (%)*
70 (13.1)	95 (17.8)	148 (27.8)	113 (21.2)	106 (19.9)	532 (99.6)

The proportion of positive, neutral and negative responses to questions concerning confidence in delivering RCs, the perceived effectiveness of care, and engagement with self-help and exercises compared with face-to-face consultations are shown in Table 3. In terms of comparative effectiveness of care nearly half of respondents felt it to be not equivalent to face-to-face care whilst just over a quarter felt it was. Conversely, confidence in delivering care and engagement with self-help advice and exercises were in comparison felt by the majority to be positive.

**Table 3:** Respondent Perceptions of Effectiveness, Confidence and Engagement With Remote Consultations (n[%])

	Strongly agree/Agree	Undecided	Disagree/Strongly disagree
I feel remote chiropractic consultations can provide effective patient care compared to a face to face consultation (533 (99.8%) responded)	142 (26.6%)	136 (25.5%)	255 (47.8%)
I feel confident in carrying out an assessment and providing information and instructions when delivering remote consultations (533 (99.8%) responded)	280 (52.5%)	115 (21.5%)	138 (25.9%)
I feel that in remote consultations I am engaging my patients more with self-help advice and exercises compared to face to face consultations (531 (99.4%) responded)	266 (50.1%)	78 (14.7%)	187 (35.2%)

**Fig. 2:**
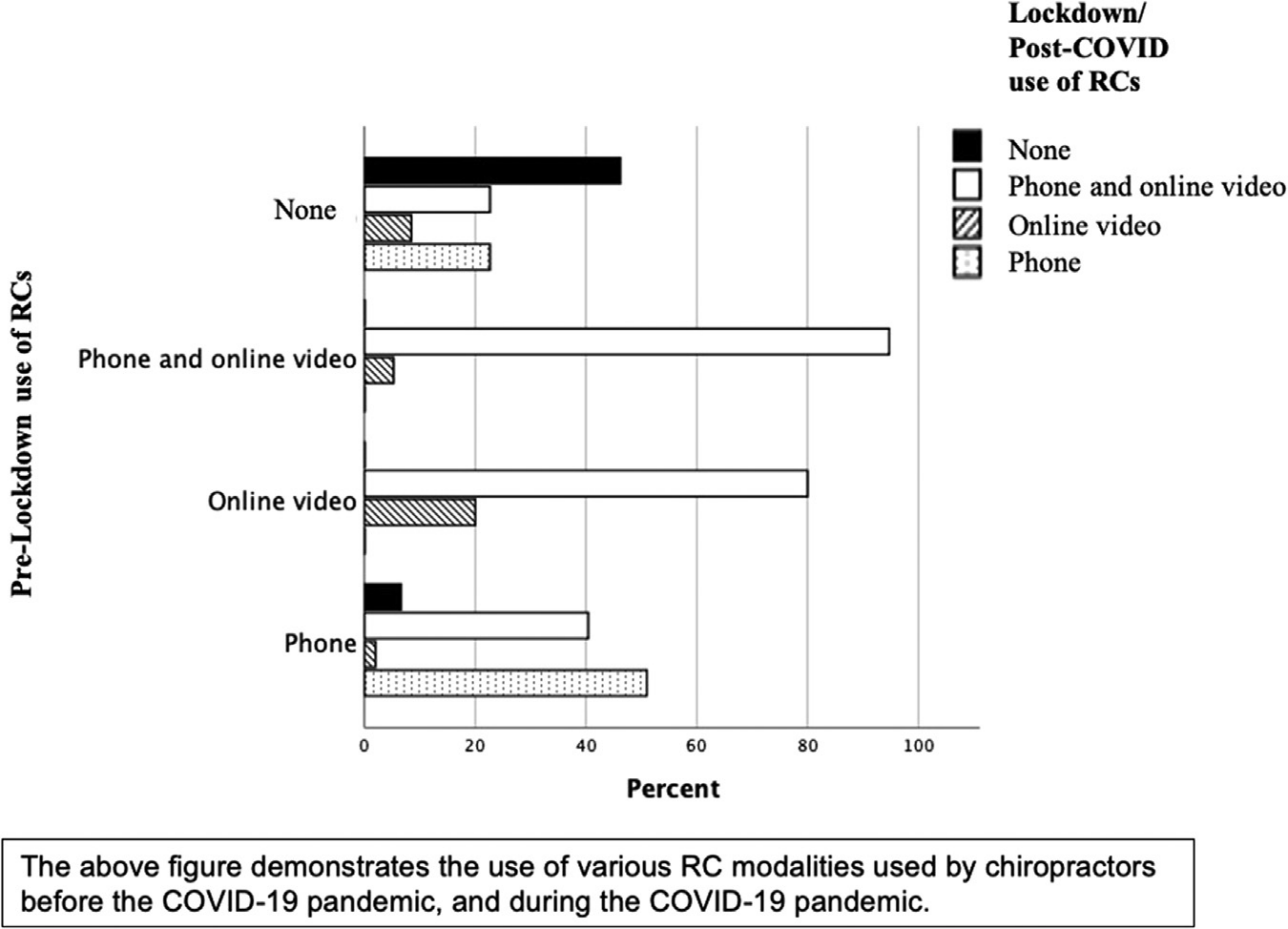
Comparison of Lockdown/Post-COVID remote consultation utilization compared with pre-Lockdown use.

Figure 2 shows the changes in preference of type of RCs from before the pandemic in the y-axis to their subsequent preference of RC type that they used during the pandemic in x-axis. Around half of the respondents not using RCs currently had not used them pre COVID-19 (Fig. 2). For those that had used RCs previously, nearly all of those using phone and video were continuing to do so. For those that used online video previously, 80% were now using both phone and video and for those using phone previously 40% had started using phone and video. There was a lower and more mixed adoption for those that had answered negatively to the use of RCs pre COVID-19.

**Fig. 3:**
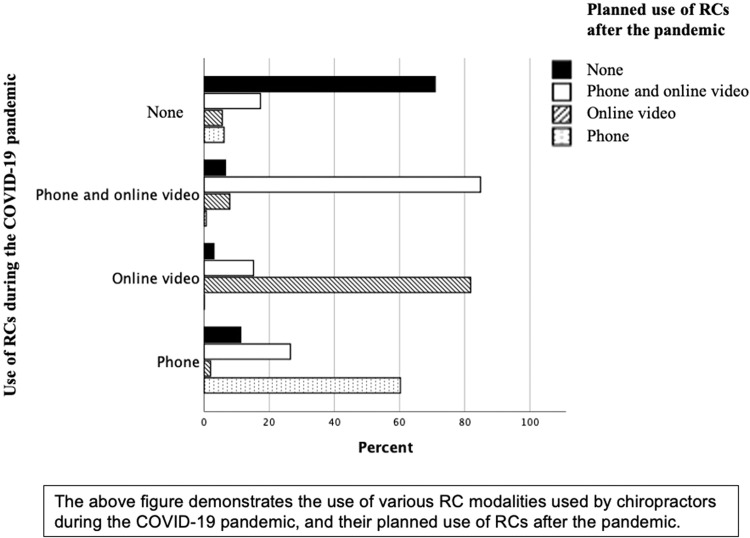
Comparison of planned use of remote consultations compared with COVID-19 pandemic use.

Figure 3 shows the changes in preference of type of RCs from during the pandemic in the y-axis to their subsequent preference of RC type that they planned to use after the pandemic in x-axis. Figure 3 indicates that for those not using any form of RC currently, over 70% indicated they were not planning to in the future. For those using phone or video or phone/video RCs, most were planning to carry on the use of the format they were currently using. Combined phone & video usage rates excluded, pre-COVID-19 rates for phone only and video only were 27.8% and 1.0% respectively, during 30.8% and 6.6% respectively, and after 19.9% and 9.2% respectively. Overall, this demonstrated a 9-fold increase in the intended use of video alone and an almost 10% decrease in phone only use.

Table 4 shows a substantive increase in the current use of RCs compared to pre COVID-19 levels where between 50-80% were not using RCs pre COVID-19. There were significant differences in current, planning and previous RCs use between age categories (Chi^2^ test for trend: *P < .*001, < .001 and < .05, respectively). Older chiropractors (60-79-year-olds) have previously used, are using and plan to continue to use phone to a greater extent than younger age groups. However, younger age groups are planning to adopt phone/video to a significantly greater extent. It is notable that around 20-45% of UK chiropractors in this study are not using, and around 20%-40% do not plan to use RCs.

**Table 4:** Pre Pandemic, Current and Planned Use of Remote Consultations by Age Categories

Age Group n (%)*	21-29	30-39	40-49	50-59	60-79
Pre Covid -19 use					
Phone	13 (22)	25 (17.9)	38 (26.8)	44 (36.7)	22 (45.8)
Video	0	1 (0.7)	2 (1.4)	2 (1.7)	0
Phone and Video	1 (1.7)	7 (5.0)	3 (2.1)	6 (5.0)	2 (4.2)
None	45 (76.3)	108 (76.6)	99 (69.7)	68 (56.7)	24 (50.0)
Current use					
Phone	14 (22.6)	38 (25.9)	41 (28.1)	41 (31.8)	26 (52.0)
Video	2 (3.2)	16 (10.9)	9 (6.2)	7 (5.4)	1 (2.0)
Phone and Video	18 (29.0)	40 (27.2)	43 (29.5)	54 (41.9)	9 (18.0)
None	28 (45.0)	53 (36.1)	53 (36.3)	27 (20.9)	14 (28.0)
Planned use					
Phone	10 (17.5)	19 (14.0)	26 (18.7)	22 (19.8)	20 (44.4)
Video	4 (7.0)	15 (11.0)	13 (9.4)	10 (9.0)	3 (6.7)
Phone and Video	26 (45.6)	55 (40.4)	48 (34.5)	58 (52.3)	9 (20.0)
None	17 (29.8)	47 (34.6)	52 (37.4)	21 (18.9)	13 (28.9)

⁎ proportions within each age group.

There was no significant difference in previous or current use between male and female practitioners. However, there was a significant difference in planning to use RCs with more females planning to use phone/video engagement (Chi^2^ test for trend: *P < .*01) (Table 5).

**Table 5:** Pre Pandemic, Current and Planned Use of Remote Consultations by Sex

Sex *n (%)*	Female	Male
Pre COVID -19 use		
Phone	72 (27.4)	70 (28.3)
Video	0	5 (2.0)
Phone and Video	8 (3.0)	11 (4.5)
None	183 (69.6)	161 (65.2)
Current use		
Phone	89 (31.8)	71 (28.0)
Video	11 (3.9)	24 (9.4)
Phone and Video	85 (30.4)	79 (31.1)
None	95 (33.9)	80 (31.5)
Planned use		
Phone	49 (19.1)	48 (20.8)
Video	14 (5.4)	31 (13.4)
Phone and Video	106 (45.1)	80 (34.6)
None	78 (30.4)	72 (31.2)

**Fig. 4:**
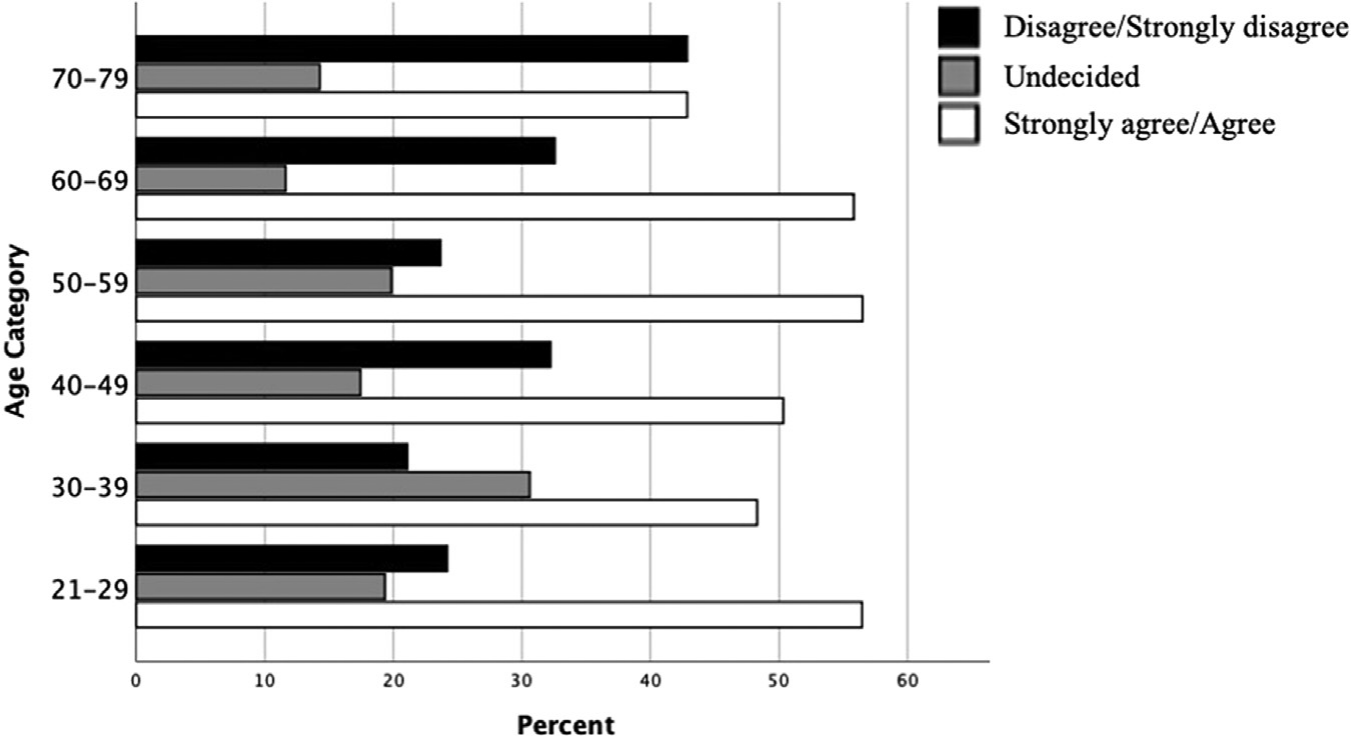
Comparison of practitioner confidence in delivering remote consultations clustered by age.

**Fig. 5:**
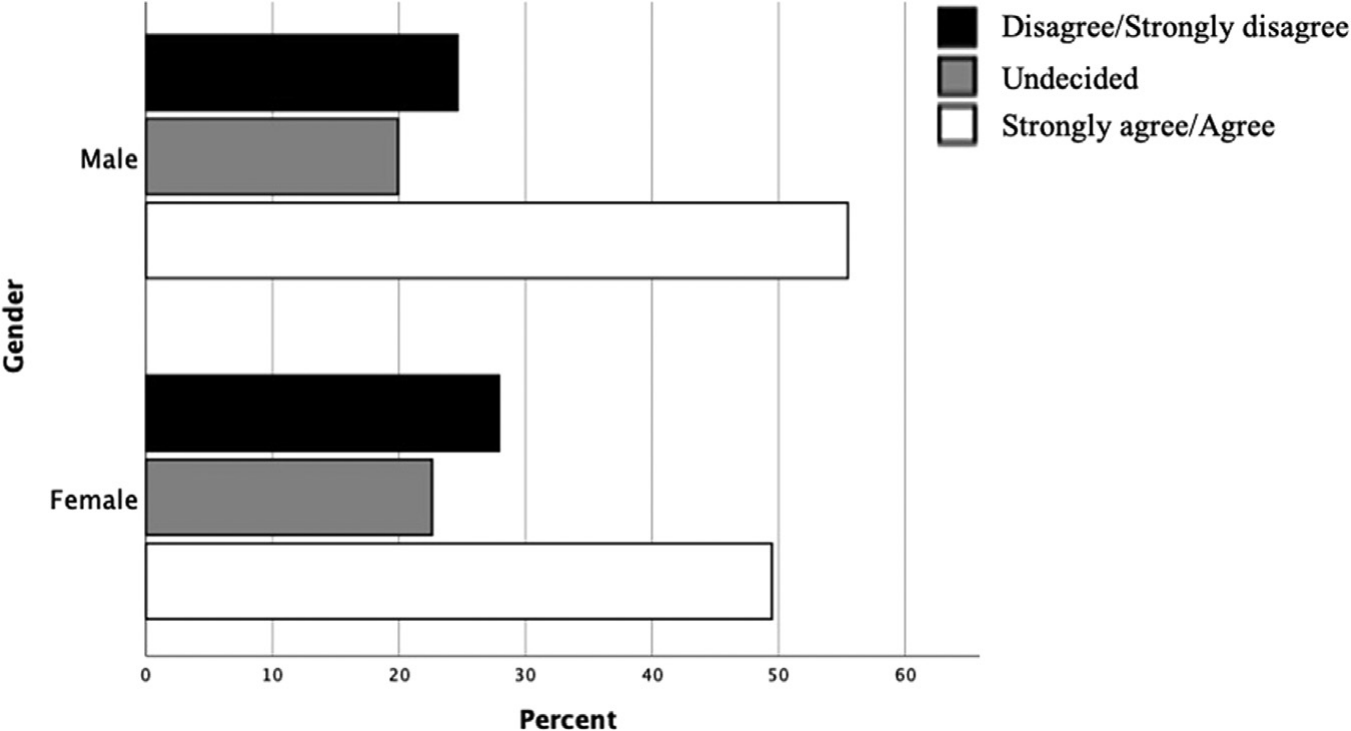
Comparison of practitioner confidence in delivering remote consultations clustered by sex.

There was no significant difference in patterns of perceived confidence to deliver RCs across age categories (Fig. 4). This is also true for years in practice as there is a high correlation with age categorisation (Spearman’s Rho; 0.79, *P < .*001). There was also no significant difference in levels of confidence between males and females (Fig. 5).

**Fig. 6:**
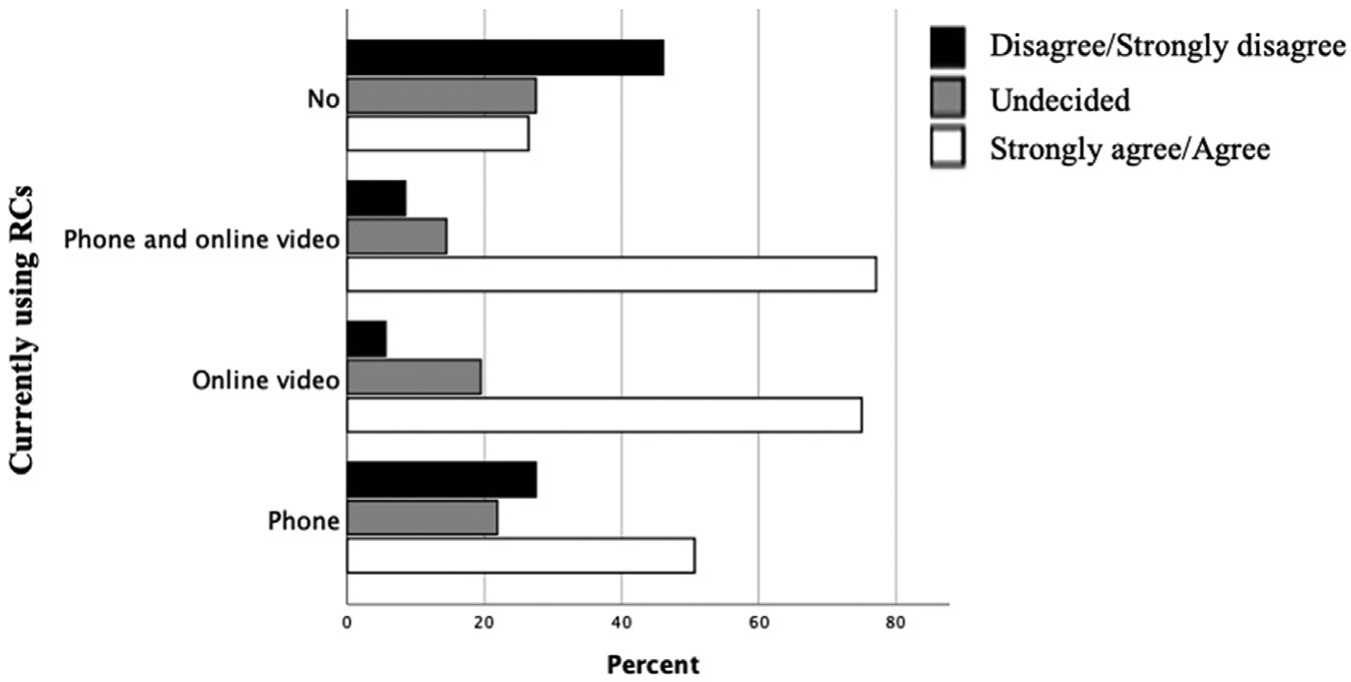
Comparison of practitioner confidence in delivering remote consultations clustered by current use.

However, there was statistically less confidence in those not currently using RCs compared with those using some form of RC with around 45% of those not using RCs disagreeing/strongly disagreeing that they felt confident to deliver RCs compared with only around 9%-27% in those that were using some form of RC (Chi^2^ test for trend, *P < .*001) (Fig. 6).

**Fig. 7:**
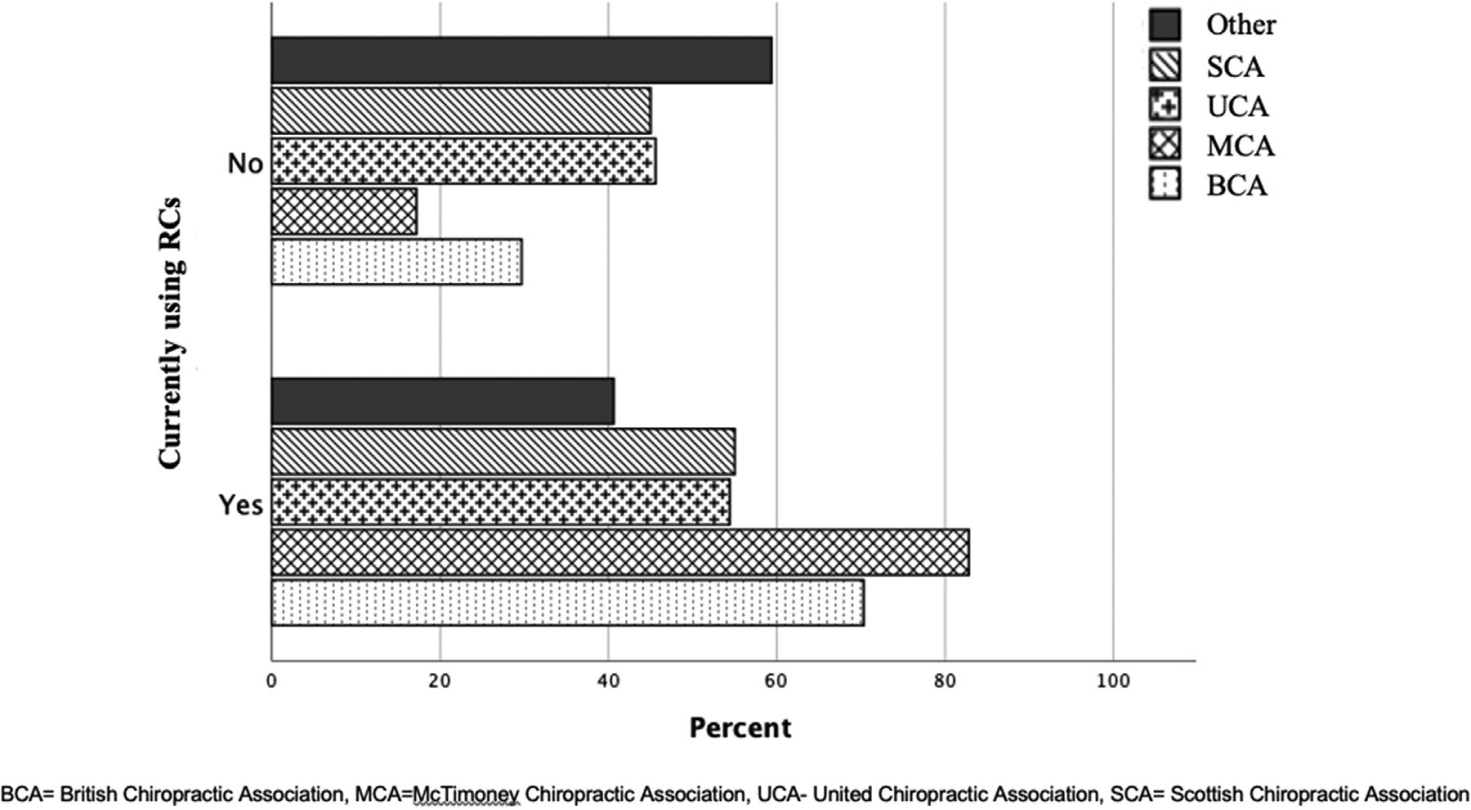
Comparison of current use of remote consultations clustered by association membership.

Generally, the majority of surveyed members of the individual associations were currently using RCs during the sampling period except those answering ‘other’ to the member association question. The largest percentages not using RCs were ‘other’, Scottish Chiropractic Association (SCA) and United Chiropractic Association (UCA) members and this was significant (Chi^2^ test for trend: <0.001) although these groups only constituted 26% of the total respondents (Fig. 7).

## Discussion

To the authors’ knowledge this is 1 of a limited number of studies where the chiropractic profession has been surveyed about the use of RCs during the early phase of the COVID-19 pandemic. This study suggests that around a third of UK chiropractors had been using some form of non-face-to-face consultations prior to the pandemic with the majority of this by telephone in an older clinician demographic. However, we cannot tell whether pre pandemic use of telephone contact with patients may have been adjunctive to a face-to-face encounter e.g. as a follow up. This is a long-standing practice amongst some in the profession, which may explain why older chiropractors’ pre COVID-19 utilization rate was higher than the younger chiropractors.^68^ Older chiropractors stated that they planned to use phone only RCs to a greater extent in the future than younger age groups, whilst younger age groups and more women are planning to use combined phone & video RCs, for age differences this may be due to habitual preferences and/or the degree of familiarity with newer technologies, however it is unknown why more women are planning to engage with this form of RCs than men.

During the first UK national lockdown the proportion of chiropractors providing RCs had risen to nearly 2 thirds of the respondents. This large increase in utilization was also seen elsewhere in the healthcare sector as well as specifically in the chiropractic profession.^69^ In 2022, Moore et al. reported the use of RCs in 6 countries; there was an increase in use of RCs by surveyed chiropractors from approximately 2%-8% pre-pandemic and 12-45% during this later phase of the pandemic (October 2020-Dec 2020).^53^ It indicated that increased use of RCs by chiropractors was associated with countries who enforced lockdowns or “stay-at-home orders”; the use of RCs in the UK during the pandemic was 45% whereas in Australia it was only 12% where limited lockdowns occurred.^53^ An American survey showed that 18.3% of US chiropractors surveyed used RCs in an early phase of the pandemic (April 2020-May 2020), the majority tended to identify with an evidence-based approach to chiropractic over a subluxation-based approach.^52^ This proportion is comparable to the 20% of Northeastern US chiropractors who were surveyed in an early phase of the pandemic (June 2020-July 2020) that reported using RCs.^51^ An analysis of U.S. Veterans Health Administration (VHA) data showed that 75% of chiropractic patient visits switched to RCs in April 2020, decreased to 17.3% from May 2020 to October 2020, and further decreased to 4.0% by March 2021; coinciding with the increase of face-to-face visits during this time period.^55^^,^^57^ In late 2019, RCs delivered by chiropractors and other complementary and integrative health therapies had been predominately conducted via phone within the VHA, whereas by March 2021 a similar use of phone and video was recorded.^57^ Although the majority of respondents to this survey were still using telephone-based RCs during this lockdown, a significant number of chiropractors chose to utilize video or a combination of video and telephone and planned to continue to use them in the future.

There has been speculation that the COVID-19 pandemic had accelerated trends in digital approaches to care already in place.^70^ However, it remains unclear whether such adoption will be perpetuated after the pandemic. A large proportion of respondents using RCs in this study stated they would probably or definitely not be offering RCs post pandemic (41.1%), with a similar proportion feeling they would definitely or very probably continue to do so (30.9%). This result suggests a clear demarcation of attitudes in the chiropractic profession concerning RCs. This was mirrored by a similar proportion of surveyed South African chiropractors, of which only 30% thought that RCs were necessary in providing chiropractic care during the pandemic and 48% did not see RCs as necessary in providing chiropractic care after the pandemic.^49^

### Perception of Effectiveness of RCs Compared to Face-to-Face Care

Nearly half of respondents (47.8%) stated their opinion that RCs could not provide patient care as effectively as face-to-face consultations, despite a quarter feeling they could. Potential reasons for this are explored in our second report.^59^ A survey of South African chiropractors in September 2020 found that 63% felt that RCs would reduce the effectiveness of chiropractic care.^49^ This compares with around 40% of GPs along with 24% of physiotherapists who perceive that “lots of” MSK problems could be successfully managed using RCs.^44^ Interestingly this perception was lower (14%) amongst physiotherapists without RC experience.^44^

### Confidence in Carrying Out an Assessment and Providing Information and Instructions When Delivering RCs

The majority of chiropractors (52.5%) stated that they were confident in carrying out an assessment and providing information and instructions when delivering RCs. Those who felt less confident were not currently using RCs, and lower confidence was reported in those using telephone consultations compared to those who used video or combined telephone/video. However, there was no significant difference in confidence across age categories raising the possibility that other factors such as familiarity with technology or experience are more important. In previous RC studies, first-hand experience of RCs alters clinicians’ initial perceptions. For example, musculoskeletal medicine physiatrists only reported comfort with use of telehealth after 10 consultations.^71^ Going forwards, training programs may be beneficial for UK graduate chiropractors, supporting upgrading of skill sets such as adapting listening, communication, and physical assessment as well as training in the use of different RC technology.^72^ Educational institutions should consider the inclusion of training in telehealth to prepare future chiropractors for use of RCs in clinical practice upon graduation. This could include training in how to adapt elements of the consultation to a telehealth environment including assessment, diagnosis, and management. Examples of delivery of telehealth consultations in an educational institution have been recently published.^73^

### Engaging Patients More in Self-Help Advice and Exercise Compared to Face-to-Face Care

Promotion of self-management strategies has been shown to have beneficial effects for MSK conditions and it is an important aspect of patient-centred care.^74^, ^75^, ^76^ The majority of chiropractors (50.1%) felt that they were engaging their patients more with self-help advice and exercise when conducting RCs compared to face-to-face care. However, 35.2% of chiropractors actively disagreed with this view. A previous study of knee osteoarthritis patients treated using RCs indicated that when physiotherapists were not able to provide hands-on treatment, they were able to focus more on interventions such as exercise, education, and self-management skills.^45^ RCs can meet a proportion of best clinical practice recommendations for MSK care and have the potential to enhance and promote aspects of active care such as self-management compared to the passive care emphasis that is typically applied in face-to-face consultations. A patient’s expectations and experience of elements of RCs vs face-to-face care could be examined in future research to determine the components that are deemed valuable by patients.^5^

### Strengths of the Study

In terms of representativeness, ratios of female to male respondents were similar to the GCC register (F = 52.4%). Previous surveys conducted in the UK profession, using random sampling (57% return rate) or convenience sampling (21 and 30% return rate) reported the proportion of females as 43%-46% or 45 and 67% respectively compared to 52% in our sample.^77^, ^78^, ^79^ Furthermore, in previous data, the proportion of membership of the UK associations (BCA, MCA, SCA, UCA and ‘Other’) was 68, 17, 4, 7 and 4% respectively.^79^ For this study these figures were 62, 12, 7.5, 12.7 and 5.8% which are broadly similar.

### Limitations of the study

However, despite previous UK surveys being comparable, these surveys themselves were either compromised by sampling methods, or were carried out over a decade ago. Therefore, using demographic benchmarking to ascertain our sample representativeness will carry limitations as they may not represent contemporary demographics. Limited response rate therefore potentially curtails the generalizability of results presented here to the wider UK profession and a degree of caution is warranted in interpreting our results.

We developed a unique questionnaire for the purposes of this study which was limited to a face validity process by 3 chiropractors only; methods of content validity, pilot testing, and creation of operational definitions were not utilized. In addition, there is the possibility of a higher response rate by those interested in or currently using RCs and those that felt strongly against the profession’s use of RCs. Selection bias may also be introduced as a result of a larger proportion of respondents from a single chiropractic association.

The questionnaire that was constructed by the authors (Supplemental file 1) for the purposes of this study was not subjected to any formal validation procedure. We took this pragmatic approach to document the emerging changes at the time at which they occurred rather than distributing a validated questionnaire after the changes in practice had occurred and risking recall bias or a change in chiropractors’ perceptions once they were allowed to return to face-to-face consultations on a regular basis. There were no validated telehealth questionnaires available that were applicable to a pandemic context with the UK chiropractic profession in mind.

Notwithstanding, this survey provides original and time unique data concerning changes in practice delivery amongst MSK clinicians during a modern pandemic.

### Future Studies

Future research might usefully explore additional cohorts including those practicing in rural areas, the elderly, and those with childcare commitments. In addition, exploration of reasons why chiropractors choose to engage or not with RCs would be important to investigate. Exploring the views towards the effectiveness of RCs, and practitioner confidence, and the promotion of self-management as well as investigating other facilitators and barriers to the use of telehealth such as financial, and technical considerations is recommended. In this respect, triangulation of the data from this survey with interviews and focus groups would enhance the validity of the findings.^80^ Future research directions might also include establishing the effectiveness of RCs alone as well as in addition to chiropractic care for long term management of patients with recurrent and persistent spinal pain.

## Conclusion

This study explored the frequency and pattern of usage of RCs by UK chiropractors and their views concerning this under-utilized approach for providing care for their patients who are accustomed to seeing chiropractors as a "hands-on" profession. Usage of RCs, including the traditional phone and the more contemporary video RCs, significantly increased during the first UK national lockdown. Our results indicate that 2/3rds of UK chiropractors who responded to the survey expressed intention of continued use of RCs beyond the COVID-19 pandemic, which necessitates further research as to how RCs can be effectively implemented to enhance patient care. Confidence in carrying out RCs and in their effectiveness is mixed, and positively, RCs allowed chiropractors to engage patients in guideline-concordant care such as active care to a greater extent. Through necessity during the COVID-19 crisis, this study showed that RCs have now become a method of care delivery for chiropractors in the UK.

## Appendix Supplementary materials

Image, application 1

## Footnotes

Supplementary material associated with this article can be found in the online version at doi:10.1016/j.jcm.2025.07.003.
