# Supplementary material for: Use of Remote Consultations by Chiropractors in the United Kingdom During the COVID-19 Pandemic: A Cross-Sectional Survey
Source: J Chiropr Med. 2025 Oct 1;24(1-4):106–18. doi: 10.1016/j.jcm.2025.07.003 (PMC12803981; doi:10.1016/j.jcm.2025.07.003)
Supplement: Supplementary file 1 [file mmc1.docx]

**Use of remote consultations by chiropractors during the COVID-19 pandemic: A UK-based cross-sectional survey**

**Supplementary file: Survey questions**

*Section 1*

*Submitting a completed survey will be taken as your consent to take part in this project and use your anonymised data.*

*"Exploration of the usage of remote consultations by chiropractors."*

Age - Which category below includes your age?

- 21-29
- 30-39
- 40-49
- 50-59
- 60-69
- 70-79
- 80 or older

Gender - What is your gender?

- Female
- Male
- Other

Are you a member of any of the following UK organisations?

- BCA
- MCA
- UCA
- SCA
- Other

How many years have you been in chiropractic practice?

- 0-1 years (new graduate)
- 2-5 years
- 6-10 years
- 11-15 years
- 16-20 years
- 21-30 years
- 31-40 years
- 41-50 years
- 51 years or more

*Section 2 – Usage of remote consultations*

Are you currently using remote consultations with your patients?

- Yes, over the phone
- Yes, via video over the internet
- Yes, both over the phone and via video over the internet
- No

Are you planning on using remote consultations with your patients?

- Yes, over the phone
- Yes, via video over the internet
- Yes, both over the phone and via video over the internet
- No

Have you used remote consultations with your patients before the COVID-19 crisis?

- Yes, over the phone
- Yes, via video over the internet
- Yes, both over the phone and via video over the internet
- No

Once the COVID-19 crisis has passed, do you believe you will continue to use remote consultations with some of your patients?

- Definitely
- Very probably
- Possibly
- Probably not
- Definitely not

*Section 3 - Views of remote consultations*

Instructions: Below is a list of statements and questions about experiences chiropractors might have with their remote consultations. Think about the experience that you have had using remote consultations, and decide which category best describes your own experience.

IMPORTANT. Please take your time to consider each question carefully.

I feel remote chiropractic consultations can provide effective patient care compared to a face-to-face consultation.

- Strongly agree
- Agree
- Undecided
- Disagree
- Strongly disagree

I feel confident in carrying out an assessment and providing information and instructions when delivering remote consultations.

- Strongly agree
- Agree
- Undecided
- Disagree
- Strongly disagree

I feel that in remote consultations, I am engaging my patients more with self-help advice and exercises compared to face-to-face consultations.

- Strongly agree
- Agree
- Undecided
- Disagree
- Strongly disagree

If you are not currently using remote consultations, or are not planning to use remote consultations, can you briefly indicate the reason(s) why. Please type "NA" in this box if you are or will be using remote consultations.

(Long answer text box).

*Section 4 - Submit your answers*

Submitting a completed survey will be taken as your consent to take part in this project and use your anonymised data.
